# Silent Infection of Highly Pathogenic Avian Influenza Virus (H5N1) Clade 2.3.4.4b in a Commercial Chicken Broiler Flock in Italy

**DOI:** 10.3390/v14081600

**Published:** 2022-07-22

**Authors:** Federica Gobbo, Claudia Zanardello, Marco Bottinelli, Jane Budai, Francesca Bruno, Roberta De Nardi, Tommaso Patregnani, Salvatore Catania, Calogero Terregino

**Affiliations:** 1 National Reference Laboratory for Avian Influenza and Newcastle Disease, Istituto Zooprofilattico Sperimentale delle Venezie, 35020 Legnaro, PD, Italy; jbudai@izsvenezie.it (J.B.); fbruno@izsvenezie.it (F.B.); cterregino@izsvenezie.it (C.T.); 2 Histopathology Department, Istituto Zooprofilattico Sperimentale delle Venezie, 35020 Legnaro, PD, Italy; czanardello@izsvenezie.it; 3 Avian Medicine Laboratory, Istituto Zooprofilattico Sperimentale delle Venezie, 37060 Buttapietra, VR, Italy; mbottinelli@izsvenezie.it (M.B.); scatania@izsvenezie.it (S.C.); 4 Veterinary Services, Local Health Unit “AULSS 9 Scaligera”, 37057 Verona, VR, Italy; roberta.denardi@aulss9.veneto.it (R.D.N.); tommaso.patregnani@aulss9.veneto.it (T.P.)

**Keywords:** HPAI emergency, clade 2.3.4.4b H5N1 HPAIV, chicken broiler, clinical signs, mortality, viral shedding, pathobiology

## Abstract

From October 2021 to January 2022, different incursions of clade 2.3.4.4b H5N1 HPAIV (Highly Pathogenic Avian Influenza Virus) occurred in several Italian regions with its main diffusion in Densely Poultry Populated Areas (DPPAs) of north-eastern Italy. Monitoring and control activities applied in the affected area clearly evidenced that turkeys and broilers were the most affected species, although several flocks of broilers at times resulted HPAIV H5N1 infected in absence of increased mortality and/or clinical signs. Thus, an approach based on sampling dead birds was adopted in the broiler sector to improve the early detection of infection; this protocol allowed us to confirm that 15 farms were HPAIV-infected with birds ready to be delivered to the slaughterhouse. The aim of this report is to describe the results of the diagnostic activities carried out in one HPAIV H5N1-infected broiler farm, three days after laboratory confirmation during the pre-movement testing without showing increased mortality or clinical signs. Thus, clinical signs, daily cumulative mortality rate (CMR), virus shedding, seroconversion, pathobiology of clade 2.3.4.4b H5N1 HPAIV as well as Avian Influenza Viruses (AIVs) environmental contamination were thoroughly examined in the infected holding. Such in-depth investigation demonstrated low infection prevalence in live birds, low environmental contamination, no seroconversion for AIVs, gross and microscopic findings compatible with systemic infection with peracute death in H5N1 HPAIV-infected birds.

## 1. Introduction

During 2021–2022, one of the most dramatic epidemics of Highly Pathogenic Avian Influenza (HPAI) virus spread thorough European Countries, affecting wild birds and captive or domestic birds. All outbreaks were attributed to HPAI virus subtype H5 of clade 2.3.4.4b, which is actually the most predominant lineage and globally distributed in Eurasia, Africa and recently also in North America [1,2]. Notably, some H5NX viruses of clade 2.3.4.4b presented genetic markers of mammalian adaptation and increased zoonotic potential; in fact, several infections have been reported in wild or captive mammals as well as in humans [2].

From October 2021 to January 2022, different incursions of clade 2.3.4.4b H5N1 HPAIV occurred in several Italian regions. The most important cluster of infection was registered in the Veneto region (north-eastern Italy) [3] where the unique combination and presence of Densely Poultry Populated Area (DPPA) and wetlands have made this part of the country one of those at highest risk of introduction of HPAIVs via migratory aquatic birds [4]. 

On October 18 2021, the first outbreak of clade 2.3.4.4b H5N1 HPAI occurred in a commercial farm of turkey meat located in the core of the DPPA.

Despite the several measures implemented by the competent health authorities to urgently mitigate the dissemination of HPAI infection (culling of affected birds, establishment of restricted and protective zone and also of a prevention zone) a great number of secondary outbreaks were observed in different poultry categories in the Veneto region, with a centripetal distribution from the turkey index case [5].

Monitoring and control activities applied in the HPAI H5N1-affected area in late November 2021 clearly evidenced that turkeys and broilers were the most affected species. Increased mortality and clinical signs were reported in turkeys, layers, commercial ducks, guinea fowls, quails and pheasant. In contrast, in the broiler sector many flocks resulted positive for H5N1 HPAIV during emergency-monitoring activities in absence of significant increased mortality, of abnormal production parameters (food and water intake and average daily weight gain) and of suspect clinical signs. An increased mortality was generally observed in these flocks 7–10 days after the laboratory confirmation of the infection.

Moreover, Avian Influenza Virus (AIV) Real-Time Reverse Transcriptase PCR (rRT-PCR) laboratory results of oropharyngeal and tracheal swabs of live birds showed that in broilers there was a lower frequency of positive samples and/or a lower viral load (Ct) if compared with other poultry categories (turkey, chicken layer, guinea fowl and quail).

All these laboratory and field-combined findings made policy makers aware of the risk of underestimating the presence of HPAIV in the broiler sector and of the need to investigate the possible role of this category in the spread of clade 2.3.4.4b H5N1 HPAIV infection. Thus, the competent authorities enforced the HPAI emergency plan by additional and compulsory testing on dead birds found in all the poultry farms. In addition, for the broiler sector pre-movement testing (delivery of birds to the slaughterhouse) had to include 60 oropharyngeal/tracheal swabs from each shed of the farm in live birds (focusing on any bird showing signs) and tracheal swab from all dead birds, if they were present. In late November 2021, such combined measures proved to be effective in detecting the HPAI infection in 15 out of 961 commercial asymptomatic broiler flocks (2%) during the pre-movement laboratory test (October–December 2021). In addition, seven of these outbreaks (1%) were only confirmed through laboratory tests carried out on dead birds. At the end of this epidemic wave, from January 2022 to March 2022, no chicken group (0%) of the 1206 tested flocks resulted positive for AIV during the pre-movement laboratory testing. The aim of this report is to describe the results of the diagnostic activities carried out in the H5N1 HPAIV-infected broiler farms, three days after laboratory confirmation during the pre-movement testing in absence of increased mortality and clinical signs.

## 2. Materials and Methods

### 2.1. Background

Following the imminent delivery of birds to the slaughterhouse, on 26 November 2021, the official veterinary authorities visited a broiler farm of 26,271 mixed-gender Ross 308 broilers (8 weeks old), divided into two different houses (nominated house 1 and house 3) and reared on the floor using wheat straw as bedding material. No vaccination for AIVs was applied in the flock. Only male broilers were present in the farm because the depletion of females was performed approximately one week before when they were sent to the slaughterhouse.

**Figure 1:**
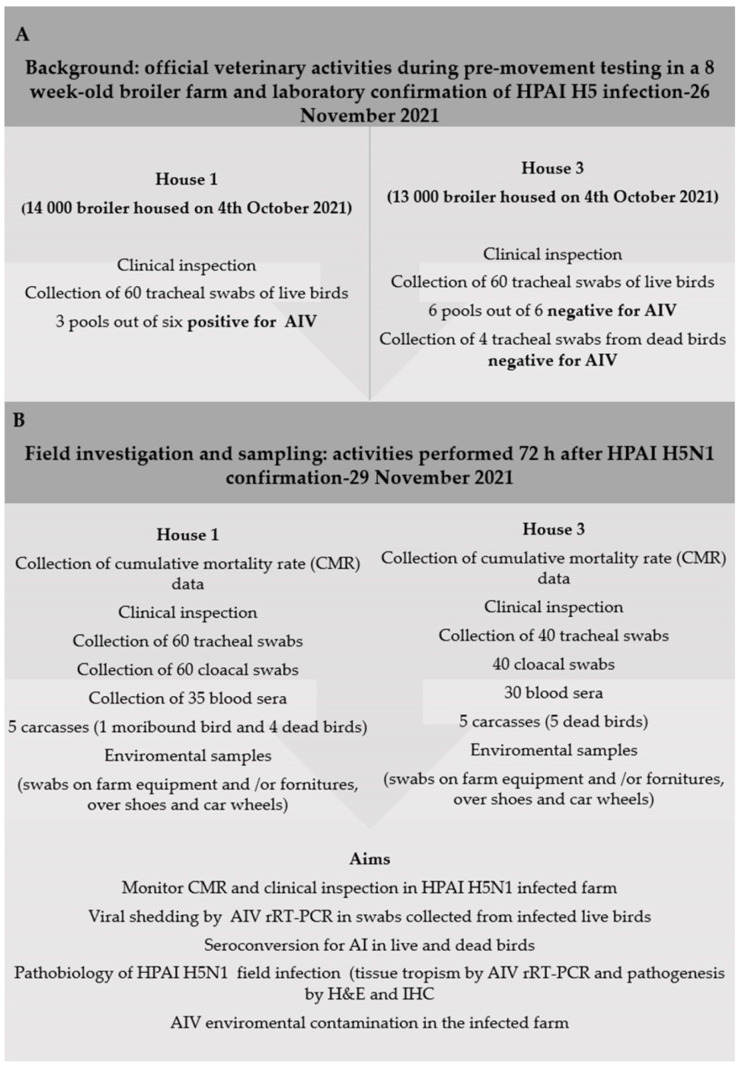
(**A**) Background of H5N1 HPAIV confirmation during the pre-movement monitoring performed by official veterinary authorities in the broiler farm. (**B**) Field activities carried out in the two houses of the affected holding for this study. AIV, Avian Influenza Virus; CMR, cumulative mortality rate; r-RT-PCR, Real-Time RT-PCR; H&E, haematoxylin and eosin staining; IHC, immunohistochemistry.

Thus, birds were clinically examined and 60 tracheal swabs were collected from live birds of both houses; only four carcasses were available in house 3 for the diagnostic analysis on dead birds through tracheal swab sampling. Thus, 124 animal swabs were sent to the laboratory, where pools of 10 swabs were performed and submitted for AIV investigation by Real-Time RT-PCR (rRT-PCR). House 1 resulted positive for AIV (three pools out of six), whereas swabs and dead birds from house 3 resulted negative (Figure 1A). Thus, the farm was promptly notified as infected with H5 HPAIV, and the movement of birds to the slaughterhouse was denied. The farm was put under restrictions until culling operations were completed. On 29 November 2021, three days after the notification of the disease and in accordance with the competent authority, new field activities were scheduled in the infected farm. Thus, different animals and environmental specimens were collected to elucidate several aspects of the HPAI infection in this asymptomatic flock of broiler (Figure 1B), to obtain data useful for the epidemiological investigation (EI) and to set up the diagnostic surveillance protocol in this specific poultry category. Thus, clinical signs, cumulative mortality rate (CMR), virus shedding, seroconversion for AIV, pathobiology of circulating H5N1 HPAI strain as well as AIVs environmental contamination were thoroughly examined. 

### 2.2. Animal Rights Statements

No supplementary permit or approvals were needed for field sampling procedures in the broiler HPAI outbreak, as the diagnostic activity was conducted as part of the emergency contingency plan for avian influenza and in accordance with the Local Competent Authorities.

### 2.3. Clinical Inspection, Sampling of Biological Specimens and Record of the Cumulative Mortality Rate (CMR) Data

On 29 November 2021, field activities of the study were carried out first in house 3 (at the time still considered not infected) and then in house 1, officially declared as being infected.

All birds were clinically examined and carefully observed for respiratory or gastrointestinal signs, neurological signs, feeding and drinking intake and litter quality. Sampling procedures on live birds were carried out through a random selection of animals, distributed along the whole house length. Thus, from each selected bird tracheal and cloacal swabs were collected; based on the time and specialists available a total of 60 birds (house 1) and 40 birds (house 3) were sampled for this study. Moreover, blood samples were also collected among selected live birds for 35 and 30 blood specimens in house 1 and 3, respectively (Figure 1B). Since one bird of house 1 was showing severe signs of respiratory distress, comb congestion, rales, lethargy and dropping wings with curling feathers, after collection of blood sample the bird was humanely suppressed by veterinarian personnel. Therefore, five carcasses for each barn were collected to be sent to the laboratory (one moribund-sacrificed bird, four deceased birds from house 1 and 5 fresh dead animal from house 3, respectively) for gross-pathology examination and collection of organs specimens for pathobiological studies of clade 2.3.4.4b H5N1 HPAIV infection in broiler.

Moreover, we asked the official veterinarians to be provided with the CMR report and available data were registered. Furthermore, environmental specimens were collected from 3 pairs of over shoes for each house, which were worn during field activities and from environmental dry swabs rubbed on inanimate surfaces. The four wheels of the car used to move between houses 3 and 1 were also sampled at the end of field activities.

### 2.4. Viral Shedding in Live Birds

All tracheal and cloacal swabs collected in live birds of houses 3 and 1 were sent to the diagnostic laboratory (National Reference Centre for Avian Influenza in Istituto Zooprofilattico Sperimentale delle Venezie) and submitted for Avian Influenza Virus Type A (AIV) rRT-PCR [6]. Briefly, swabs were individually moved into single tubes, containing a sufficient amount of PBS (with antibiotics) to ensure their full immersion (1 mL), thus swabs suspensions were vortexed for 30 s and centrifuged for 2 min at 15,000× *g* and the supernatant harvested for RNA extraction.

RNA extraction and Real-Time RT-PCR for AIV: nucleic acid extraction was performed using QIAsymphony DSP Virus/Pathogen Kit (Qiagen, Hilden, Germany) or MagMAX Pathogen RNA/DNA Kit (Applied Biosystems, Waltham, MA, USA) on the QIAsymphony SP instrument (Qiagen) and KingFisher Flex Magnetic Particle Processor (Thermofisher Scientific, Waltham, MA, USA), respectively. Amplification reaction was assembled with the AgPath-ID One-Step RT-PCR Reagents (Applied Biosystems), using CFX 96 Deep well Real-Time PCR System, C1000 Touch (Biorad, Hercules, CA, USA) as platform.

### 2.5. Serological Analyses

A total of 40 blood samples were harvested from live (35 sera) and dead birds (5 sera) of house 1, while a total of 35 blood samples were harvested from live (30 sera) and dead bird (5) in house 3.

Samples were sent to the laboratory for detection of antibodies against the internal nucleocapside of type A Influenza, using a commercial competitive multi-species ELISA following the manufacturer’s instruction (IDVet^®^, Grabels, France).

### 2.6. Study of Tissue Tropism and Pathogenesis on Carcasses

Ten male broiler carcasses (8 weeks old) were submitted for gross-pathology examination and for collecting several organs (trachea, lung, spleen, duodenum, pancreas, cecal tonsils, *Bursa of Fabricius* and brain). Thus, each organ was collected individually using sterile scissors and forceps to avoid any contamination and divided into two different aliquots, one for the study of tissue tropism by AIV rRT-PCR [6] and the remaining one for tissue fixation in 10% neutral buffered formalin in order to perform histology and immunohistochemistry. Briefly, 150–200 mg of organs (approximately 1 cm^2^) was added into a sterile tube, containing a stainless-steel bead; thus, 450–600 μL of PBS (with antibiotics) was added to the tube to obtain a 1:4 *w*/*v* dilution and homogenize at 30 Hz for 3 min using TissueLyser II (Qiagen); the organ suspension was centrifuged for 2 min at 15,000× *g* and supernatants were harvested. Finally, the organ supernatants were submitted to RNA extraction and Real-Time RT-PCR for AIV as previously described. 

Briefly, the same organ samples were routinely processed for histology and 4 μm sections were stained with haematoxylin and eosin (HE). Immunohistochemistry (IHC) was performed on 3 μm sections from each tissue positive to molecular analysis (PCR positive) using the Discovery Ultra IHC/ISH research platform immunostainer (Roche Diagnostics Corporation, Indianapolis, IN, USA). The antigen retrieval was performed using the commercial ready to use solution Protease 2 (Roche, Mannheim, Germany), at 36 °C, for 12 min. The slides were incubated with the Monoclonal Antibody to Influenza A Virus (Meridian bioscience, Cincinnati, OH, USA) targeting the nucleoprotein of influenza type A viruses applied at 1:100 dilution, for 80 min at room temperature. A ready to use antibody diluent including casein (Blocker 1, Roche, Mannheim, Germany) was added as a background blocking protein, at room temperature, for 12 min. An ultraView Universal DAB Detection Kit (Roche, Mannheim, Germany) was used as detection system. A positive control from a lung of an Influenza A positive chicken and a negative control from a kidney of an Influenza A negative chicken were included in each run. The number of immunolabelled cells, detected as nuclear staining, was assessed semi quantitatively in each individual organ and scored as follows: − (negative), −/+ (barely/occasional presence of immunolabelled cells), ++ (small number of cells), +++ (moderate number of positive cells), ++++ (numerous positive cells), +++++ (widespread immunolabelling).

### 2.7. Environmental Contamination

On 29 November, also environmental samples were collected in both houses by rubbing inanimate surfaces with dry swabs that were for being submitted to the laboratory for Avian Influenza Virus Type A (AIV) rRT-PCR [6]. Thus, in house 1 and 3 we sampled the handles of the wheelbarrows used for sampling and 3 pairs of over shoes worn during sampling activities; moreover, additional samples were collected from house 1 rubbing the wheelbarrow used for the daily collection of dead birds and from the handles of the house entrance as well as of the freezer device used for storing the dead birds in house 3. Finally, swabs were also rubbed from each wheel of the service car used by veterinarians during the field activities.

These samples were submitted to the laboratory, where the swabs and over shoes were moved into a sterile box and PBS (with antibiotics) was added to obtain a final 1:4 *w*/*v* dilution. The samples were vortexed thoroughly for 30 s and centrifuged for 2 min at 15,000× *g*. Furthermore, supernatant was harvested and immediately filtered via a membrane filter (0.45 µm pore size). Finally, the filtered supernatants were submitted to RNA extraction and Real-Time RT-PCR for AIV as previously described.

## 3. Results

### 3.1. Clinical Inspection of Birds, Record of the CMR Data and Sampling

**Figure 2:**
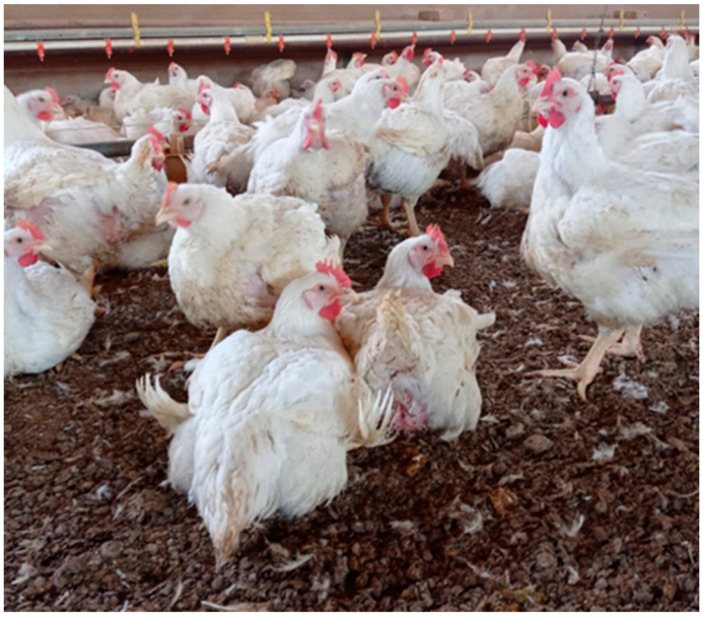
A picture of live birds in normal health status of the infected house 1. A laying bird shows dropping wings and curling feathers.

Birds of houses 1 and 3 appeared in good general health during clinical inspection and no notable respiratory, gastrointestinal or neurological signs were observed. Birds showed normal drinking and food behaviours, the only relevant signs being mild cutaneous cellulitis and breast blister in few birds. Moreover, only in house 1 aspecific and mild respiratory signs (conjunctivitis, rales, serous nasal discharge) or dropping wings with curling feathers were observed in a small number of birds (Figure 2).

**Figure 3:**
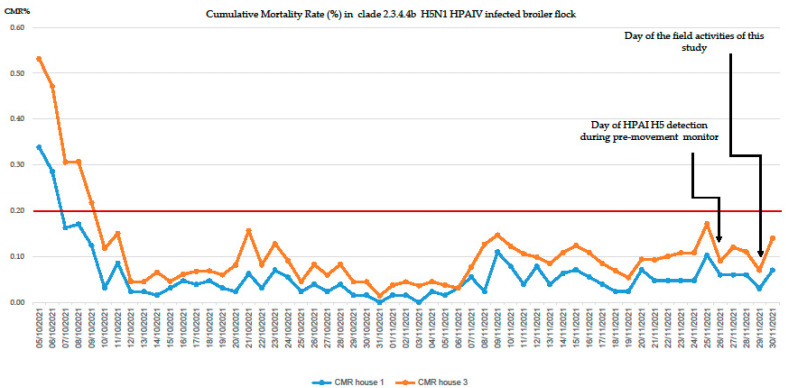
CMR, Cumulative Mortality Rate (%) of infected holding during the entire production cycle. House 1 CMR data (blue) and house 3 CMR (orange). The red bar indicates the value of 0.2% CMR-early warning of suspect AIV infection.

The elaborated data of the CMR of houses 1 and 3 are available below in Figure 3. 

It is interesting to note that despite abnormal mortalities occurring soon after the day of housing of the broiler chicks (on 4 October 2021), no daily cumulative mortality (greater of 0.2) was reported during the whole production cycle in both houses, especially in the week before H5N1 HPAI confirmation. On the day of the field investigation on 29 November 2021, one moribund bird and four dead birds were collected from house 1, and five dead birds from house 3. Subsequent epidemiological investigation pointed out the mortality rate was maintained under 0.2% until 30 November 2021. Higher and abnormal CMR were reported in both houses only eight days after the laboratory confirmation of HPAI infection (from 4 December 2021) (personal communication).

### 3.2. Viral Shedding in Live Birds

In house 3, no tracheal or cloacal swab from 40 live birds provided positive results by AIV rRT-PCR, meaning that this house could still be considered negative on the basis of the intra vitam sampling. In contrast, in the infected house 12 birds out of the 60 examined (20%) resulted H5N1 HPAIV positive. The results of the viral load (Ct values of AIV rRT-PCR), in tracheal and cloacal swabs of infected live birds of house 1 are reported in Table 1.

Only cloacal shedding was observed in two birds, whereas five animals were positive only in tracheal swabs. Moreover, five birds showed simultaneous respiratory and cloacal viral shedding, and among them two broilers (Table 1. bird 6/BRL24 and bird 7/BRL30), both seeming symptomatic with mild respiratory signs, presented a very high load of viral excretion. Furthermore, one bird, showing dropping wings with curling feathers, was found positive in the trachea and cloaca (Table 1. bird 3/BRL8). All the other birds tested positive for H5N1 HPAIV did not report any notable clinical signs during animal handling and sampling procedures.

### 3.3. Serology for AIV by Competitive ELISA Method

All the 65 blood samples collected from live birds of house 1 and 3 provided negative results for antibody detection of Avian Influenza Viruses by ELISA (IDVet^®^), as well as the 10 sera blood samples obtained from cardiac clot of all carcasses. Thus, all birds from house 1 shedding H5N1 HPAIV, in different viral loads and through respiratory and/or cloacal elimination, were negative by ELISA 3 days after confirmation of the HPAI outbreak.

### 3.4. Study of Tissue Tropism and Pathogenesis (Pathobiology of HPAI Infection)

**Figure 4:**
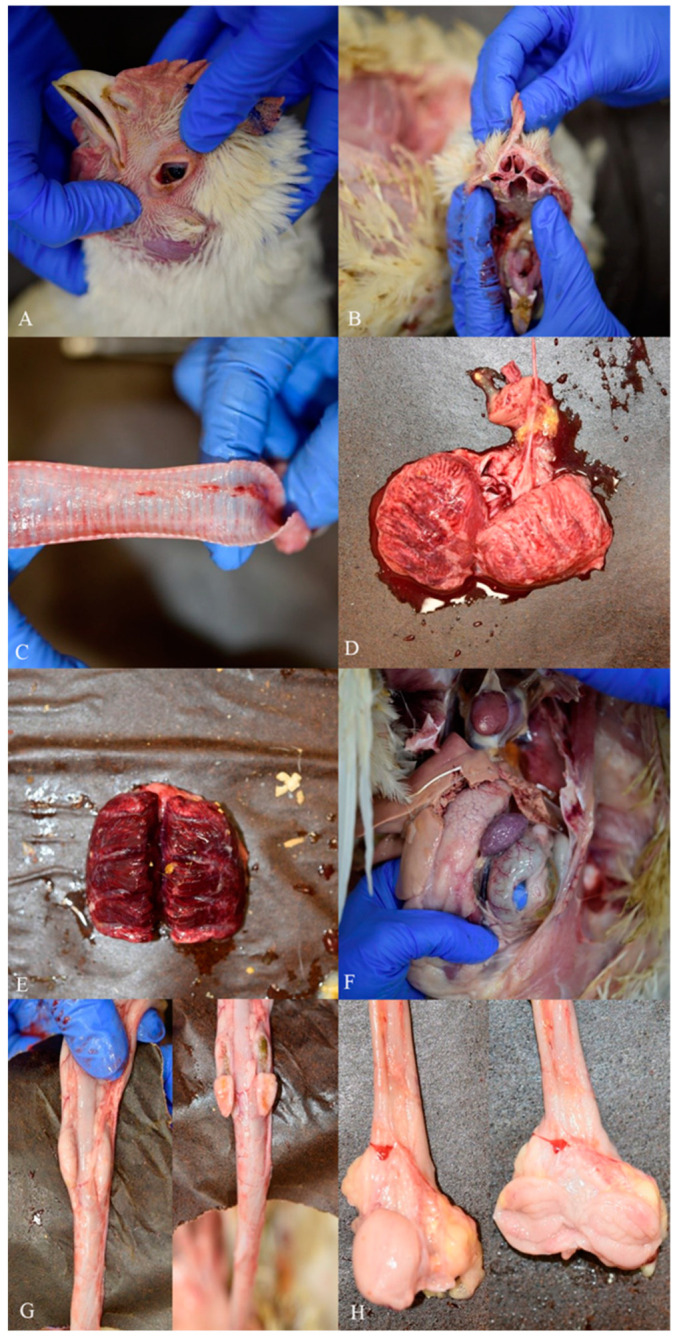
Main gross-pathology findings in infected birds of house 1. (**A**) Eye: moderate to severe conjunctival congestion and mild serous conjunctivitis. (**B**) Nasal cavities: mild hyperemia of nasal mucosa with serous debris not associated with diffuse oedema. (**C**) Trachea: hyperaemia, petechial haemorrhages and serous-catarrhal luminal exudation are visible on the mucosal surface. (**D**,**E**) Lungs: bilateral, moderate to severe, focally extensive to diffuse, pulmonary consolidation with edema, congestion and haemorrhages. (**F**) Spleen: splenomegaly with diffuse, miliary whitish spots. (**G**) Cecal tonsils: external (**left**) and cut (**right**) surface. Moderate hyperplasia with multifocal, red to light orange, small patches on the cut surface. (**H**) *Bursa* of *Fabricius*: external (**left** figure) and inner (**right** figure) surface. Moderate hyperplasia and mild mucosal oedema.

Gross pathology examinations of the five carcasses of house 1 provided comparable necropsy findings. Birds were in good state of nutrition and hydration presenting feed in crop or gizzard; moreover, no soiled vent or cutaneous congestion/haemorrhages were observed among birds. Main pathological lesions were distributed to the respiratory apparatus with conjunctival congestion and serous conjunctivitis (Figure 4A), nasal turbinates were generally mildly hyperaemic but not oedematous, showing serous discharge only in some birds (Figure 4B). Signs of tracheitis were observed in all the birds and were associated with diffuse hyperaemia of *mucosae*, multifocal petechial and serous or serous-catarrhal exudation in the lumen of the organ (Figure 4C). Lungs were bilaterally and severely affected showing marked congestion and diffuse oedema of the parenchyma (Figure 4D,E). Notably, all birds presented enlarged spleen associated with diffuse and miliar whitish spot lesions (Figure 4F). Serous-catarrhal necrotic enteritis associated with a marked vessel congestion of the gut wall was also a common finding among the examined birds.

Pancreas parenchyma did not show notable lesions despite focal areas of necrotic pancreatitis in two birds. Generally, cecal tonsils of positive birds showed hypertrophy but in absence of specific lesions related to AIV infection (Figure 4G). *Bursa* of *Fabricius* were notable in size despite the age of birds and presented mild sign of diffuse oedema of the mucosa with focal hyperaemic areas (Figure 4H). Carcasses from house 3 did not present any notable lesions, despite one bird showing serous-catarrhal tracheitis, severe lung congestion and oedema, and splenomegaly with diffuse miliar whitish lesions. Other carcasses showed enlarged liver, necrotic enteritis and abdominal and thoracic aerosacculitis with signs of neovascularization of the thoracic air-sacs. 

**Figure 5:**
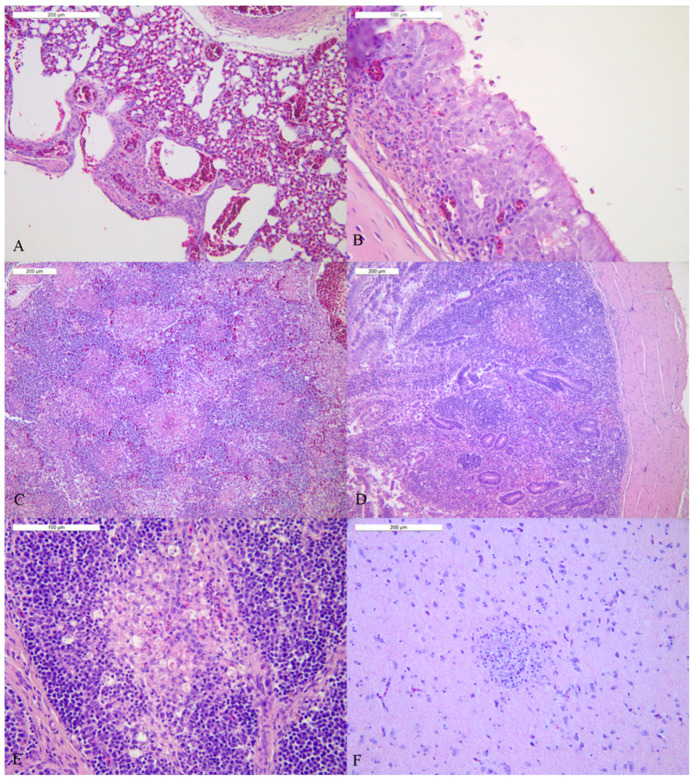
Histopathology of organs of broiler of house 1 (HE): (**A**) Lung: congestion of the parenchyma and small necrotic parabronchial area admixed with few heterophils. (**B**) Trachea: foci of necrosis admixed with heterophils and few lymphoplasmacells. (**C**) Spleen: severe multifocal to coalescing necrosis with heterophilic infiltration. (**D**) Cecal tonsils: multifocal necrosis of the mucosa admixed with heterophils and diffuse lymphoplasmacytic infiltration of the *lamina propria*. (**E**) *Bursa of Fabricius*: focal depletion of lymphocytes in the medulla with necrosis and heterophils, (**F**) brain: focal necrosis with activated microglial cells.

Histopathology of the organs of the birds of house 1 presented comparable lesions (Figure 5), with the exception of broiler 2 that had mild or no histological lesions. Tracheas showed mild to moderate foci of lymphoplasmacytic infiltration in the mucosa and foci of cellular necrosis and loss of cilia; necrotic cells appeared as vacuoles containing debris and were admixed with few heterophils. The lungs were congested with multifocal interstitial to parabronchial necrosis admixed with heterophils and macrophages in two of the three animals. The spleen of four birds presented moderate to severe and multifocal to coalescing necrosis with heterophilic infiltration; moreover, the splenic follicles were multifocally reactive. The *lamina propria* of the intestine was diffusely infiltrated by lymphoplasmacells and mild necrosis of the mucosa was observed. Only one animal showed a focal area of necrosis admixed with heterophils in the *muscularis mucosae*. Except for one broiler that showed rare foci of necrosis in the pancreas, other animals did not present notable pancreatic lesions. Cecal tonsils showed moderate to severe multifocal to coalescing necrosis of the mucosa with villus atrophy. The *Bursa* of *Fabricius* presented multifocal mild to moderate loss of lymphocytes in both the cortexes and medullae of the follicles, sometimes associated with necrosis and interstitial oedema and heterophilic infiltration. Finally, in the brain sections multifocal microfoci of necrosis with activated microglial cells were detected in four out of five birds.

In contrast, only one out of the five carcasses from house 3 presented significant lesions in all the organs, while the other animals showed mild or no lesions at all. This one bird presented diffuse congestion of the lung with mild and multifocal parabronchial necrosis; the trachea showed mild and rare foci of lymphoplasmacytic infiltration in the mucosa and foci of cellular necrosis. The spleen was multifocally necrotic and infiltrated by heterophils. The mucosa of the cecal tonsils and the intestine was necrotic and lymphoplasmacytic infiltration was detected in the *lamina propria*. Foci of mild loss of lymphocytes in the follicles of the Bursa. Histologically the brain showed multifocal microfoci of necrosis associated with activated microglial cells.

A scoring system for immunohistochemical staining was set to evaluate the distribution of AIV nucleoprotein antigen in H5N1 HPAIV-infected tissues screened by AIV rRT-PCR, and their combined results are available in Table 2 and Table 3 for house 1 and 3, respectively.

**Figure 6:**
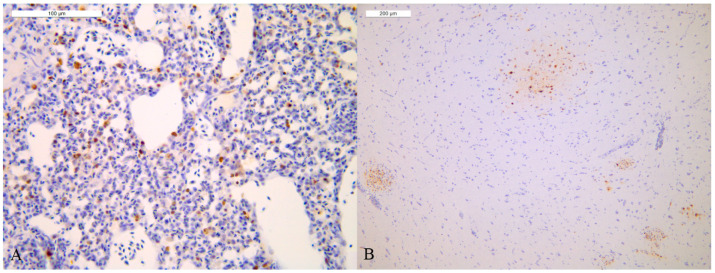
Lung and brain of one broiler of house 1 (IHC) (**A**) Lung: moderate number of positive pneumocytes and macrophages detected as nuclear brown staining (+++ score), (**B**) brain: moderate number of positive neurons and microglial cells detected as nuclear brown staining (+++ score).

All carcasses of house 1 (one moribund sacrificed bird and four freshly dead birds) presented a systemic infection with H5N1 HPAIV. The moribund bird (BRL1) was the one with the highest quantity of viral RNA in all the examined organs. All the bird except one (BRL 2) were IHC positive. The lungs and brains obtained the highest IHC score (Figure 6) as well as the lowest Ct values in AIV rRT-PCR. In addition, a general infection of lymphoid tissues (spleen, cecal tonsil and *Bursa of Fabricius*) was observed in all the birds of the infected house. Interestingly, four birds out of five showed a high IHC score in the pancreas and a high viral load (Ct) in absence of notably macroscopic and microscopic lesions. Finally, the predominant positive IHC cell types are epithelium cell, endothelium, macrophages, neurons and glial cells.

**Figure 7:**
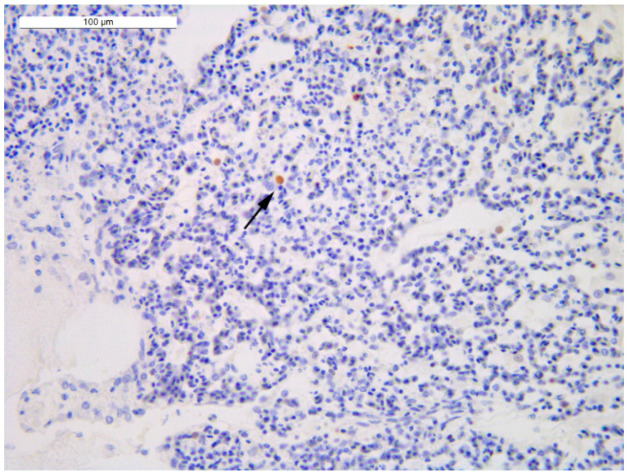
Lung of the broiler 4 of house 3 (IHC): barely/occasional presence of immunolabelled pneumocytes (black arrow) detected as nuclear brown staining (−/+ score).

All carcasses of house 3 were daily-fresh dead birds. Interestingly, BRL 4 (Table 3), the only bird of house 3 presenting either macroscopic or microscopic lesions, showed H5N1 HPAIV systemic infection with a viral load comparable to the one reported in house 1, but with a barely/occasional presence of immunolabelled cells (Figure 7).

### 3.5. Environmental Contamination

Some environmental samples collected only from house 1 provided positive results by AIV rRT-PCR, showing Ct values higher than the positive threshold cut-off (>36.00 Ct). In detail, two pools of over shoes out of three presented a very low viral load (37.4 Ct and 38.8 Ct) as well as the swab taken from rubbing the handles of the wheelbarrow used during sampling activities (36 Ct). No environmental samples collected in house 3 resulted positive for AIV genome detection and neither did the samples obtained from the wheels of the service car used by veterinarians during the field activities. All these results suggest a low viral load in the infected house both on inanimate surface and in the litter bedding (over shoes).

## 4. Discussion

The results of this study allowed us to acquire a realistic field picture of the “silent infection behaviour” of several clade 2.3.4.4.b H5N1 HPAIV outbreaks occurred in the broiler sector in Italy in the 2021–2022 HPAI epidemic. Differently from what other authors observed [7], the monitoring of abnormal CMR (greater than 0.5) and/or notable clinical signs were neither alone nor when combined a successful tool for the early detection of HPAI virus infection in several broiler flocks hit by the recent Italian epidemic.

Similar clinical patterns observed in the most recent HPAI Italian epidemic have been reported in broiler also by other European countries during different 2021–2022 H5NX HPAIV incursions; more in detail, 24 out of 230 (10.4%) European outbreaks occurred in commercial broilers that did not show mortality and/or clinical signs [2]. Epidemiology investigation conducted in the farm by the Local Health Unit Veterinary Services confirmed abnormal mortality began on 3 December 2021; thus, only eight days later the farm was confirmed HPAI infected. This finding combined with the estimated temporal dynamic of infection of house 3 let us suppose that virus introduction in the holding occurred at least 11 days before the onset of clinical signs and increased mortality. In house 1, 12 live birds out of 60 that tested positive for HPAI (20%) showed low viral loads in trachea and/or cloaca in absence of notable clinical signs; in addition, the constant finding of feed in crops or gizzards of the examined HPAI positive carcasses suggested normal feed intake and sudden death. Moreover, higher and recurring respiratory shedding rather than cloacal shedding were observed in positive live birds of house 1, as reported from other authors in experimental clade 2.3.4.4b H5 HPAIV infection of chickens [8,9]. Furthermore, the low level of viral contamination from the environmental samples of the infected house, as well as the low prevalence of infected birds suggest a low morbidity rate but a high lethality rate with the sudden death of the birds in absence of premonitory clinical signs. In our study, it is worth mentioning that the tracheal and cloacal swabs collected from 40 live birds of house 3 did not evidence any signs of infection; only laboratory results obtained from dead birds allowed us to demonstrate that this house had been infected with H5N1 HPAIV. The high and diffused level of viral load in the organs of 5/10 carcasses (four from house 1 and one from house 3), combined with macroscopic/microscopic pathology and IHC immunolabelling, are consistent with a systemic infection with multi-organs failure. Therefore, the absence of specific antibody response in all the tested birds of house 1 could be due to the fact that positive birds may have no time to seroconvert given the rapid onset of HPAI general infection and incur in a sudden death.

Gross lesions of the spleen were regularly present in all the infected dead birds, and these macroscopic findings were also recurrently observed in other poultry categories (turkey, layer, guinea fowl and pheasant) during the 2021–2022 HPAI epidemic in Italy (data not shown). Histopathology of the spleen was comparable among positive broilers and generally associated with severe tissue damage with macrophages and endothelium resulted as predominant IHC positive cell type. In addition, the cecal tonsils and *Bursa* of *Fabricius* presented high viral loads as well as anatomical and functional impairment, suggesting a possible concurrent status of immunodeficiency in the affected birds. Pathobiology results obtained in this study from the dead commercial broilers showed that 2021–2022 clade 2.3.4.4b HPAIV H5N1 infection involved different organs with a specific respiratory tissue tropism for lung, endothelium and brain. These findings confirm the results achieved by other authors in field and experimental studies with this pathogen [10,11].

The likely mechanisms responsible of this “silent infection behaviour” in broiler flocks observed in Italy during the last H5N1 HPAI epidemic emergency, with a low level of prevalence and low viral load in most examined samples of live birds, are still unknown. A systemic review and meta-analysis of the virus shedding of avian influenza viruses [8] considered different explanatory variables for the viral shedding of AIVs in poultry species such as pathotype (HPAI/LPAI), shedding route, virus origin, inoculation route, age and chicken categories; it is reported that low viral shedding in chicken can be due to the avian host from which the virus originated. Moreover, some avian genes involved in resistance to clade 2.3.4.4b H5N8 HPAIV in commercial broilers (Ross 308 Broiler) and six local Spanish chicken breeds have recently been experimentally investigated [12] and PLAU, VCAM, TNFRSF1-1A and PGF gene expression, which are mainly involved in inflammation response, resulted downregulated in resistant chickens.

During the Italian HPAI emergency of 2021–2022, an approach generally adopted in duck and geese farms to improve the early diagnosis of the infection based on the sampling of dead birds was implemented in the broiler sector in compliance with the EFSA AHAW Panel 2021 scientific opinion [13]. According to this document, the recommended surveillance strategy to be adopted in poultry species/categories that may not show significant clinical signs when infected with HPAIV, is based on the weekly clinical examination of flocks and collection of carcasses/samples of dead birds from each shed on the day of sampling. Another part of carcasses/samples should be uniformly collected in the holding from frozen carcasses found dead in previous 3–5 days.

On the basis of our findings, this protocol could be extended to broilers and to this purpose, specific guidelines (weekly pool sampling or “bucket sampling”) were included in the page of the AI-ND EURL website dedicated to sampling and diagnostic protocols [14].

According to our results, the samples originating from dead birds resulted a more rapid and sensitive tool for the early detection of clade 2.3.4.4b H5N1 HPAIV infection in the broiler sector, at least in those holdings presenting normal MR and a good health status of the flocks. Since no seroconversion was demonstrated in all the sera by ELISA, the application of serology does not seem to be an efficient diagnostic approach for monitoring or control activities in the broiler category. Finally, during the H5N1 HPAI epidemic emergency the measure adopted to include diagnostic samples originating from the dead animals for pre-movement purposes, allowed us to confirm 15 broilers farms being HPAIV-infected when ready to be delivered to the slaughterhouse; this made it possible not to move thousands of potentially infected live birds across the DPPAs of the Veneto region.

## 5. Conclusions

In recent years, we have observed globally an increased spread of clade 2.3.4.4b H5NX HPAI viruses, following the ability of these viruses to infect migratory wild birds and in many cases asymptomatically. Update studies on the evolution of circulating HPAI viruses in the wild reservoirs as well as their behaviour in each poultry species/categories should be encouraged to obtain deeper scientific data on their potential or epidemiological role in HPAI epidemics. This knowledge could also provide important scientific evidence to set up in a more efficient way control strategies and surveillance activities in HPAI emergency by developing specific monitoring protocols for each poultry category.

## Figures and Tables

**Table 1:** Results of rRT-PCR for AIV of samples collected in 12 positive live birds out of 60 (20%) in house 1.

House 1 Progressive Bird N°	Broiler (BRL) Identification N°	Tracheal Swab AIV rRT-PCR (Ct)	Cloacal Swabs AIV rRT-PCR (Ct)
**1**	BRL 6	neg	33.45
**2**	BRL 7	39.05	35.65
**3**	BRL 8	35.6	39.32
**4**	BRL 9	neg	34.7
**5**	BRL 10	34.97	36.04
**6**	BRL 24	25.79	28.46
**7**	BRL 30	21.27	23.77
**8**	BRL 47	38.46	neg
**9**	BRL 51	32.89	neg
**10**	BRL 52	34.95	neg
**11**	BRL 54	31.51	neg
**12**	BRL 55	34.18	neg

N°, number; BRL, broiler; AIV, Avian Influenza Virus; rRT-PCR, Real-Time RT-PCR; Ct, cycle threshold, neg, negative.

**Table 2:** AIV rRT-PCR (Ct) results and IHC score of organs collected from dead birds of house 1.

House 1	BRL1		BRL2		BRL3		BRL4		BRL5		
**Organ**	**rRT-PCR** **(Ct)**	**IHC Score**	**rRT-PCR** **(Ct)**	**IHC Score**	**rRT-PCR** **(Ct)**	**IHC Score**	**rRT-PCR** **(Ct)**	**IHC Score**	**rRT-PCR** **(Ct)**	**IHC Score**	**Predominant IHC Positive Cell Type/Tissue**
**Lung**	17.6	+++	34.01	−	21.06	+++	18.77	+++	18.43	+++	Pneumocytes, endothelium
**Trachea**	18.9	+	27.89	−	21.67	++	21.96	++	19.77	+++	Epithelial cells, macrophages, endothelium
**Spleen**	19.07	+	36.13	−	19.81	+++	22.08	++	23.32	++	Macrophages, endothelium
**Duodenum**	18.35	++	31.32	−	22.39	+	21.36	+	20.09	+++	Cellular debris in the crypts, epithelium, endothelium and plexi
**Pancreas**	17.17	+++	33.57	−	22.19	++	22.21	+	18.73	+++	Acinar necrotic epithelium
**Cecal tonsil**	16.81	+++	25.32	+	neg	n.d.	22.46	++	18.29	++	Cellular Debris in the crypts, epithelium, necrotic area, endothelium
***Bursa of*** ***Fabricius***	18.19	+++	32.91	−	20.25	+	21.71	++	15.15	++	Macrophages
**Brain**	13.85	+++	34.6	−	19.83	++	17.2	+++	18.94	+++	Neurons, glial cells, ependymal

BRL, broiler; rRT-PCR, Real-Time RT-PCR; Ct, cycle threshold; IHC score, − (negative), −/+ (barely/occasional presence of immunolabelled cells), ++ (small number of cells), +++ (moderate number of positive cells), ++++ (numerous positive cells), +++++ (widespread immunolabelling); neg, negative; n.d., not done.

**Table 3:** AIV rRT-PCR (Ct) results and IHC score of organs collected from dead birds of house 3.

House 3	BRL1		BRL2		BRL3		BRL4		BRL5		
**Organ**	**rRT-PCR** **(Ct)**	**IHC Score**	**rRT-PC** **(Ct)**	**IHC Score**	**rRT-PCR** **(Ct)**	**IHC Score**	**rRT-PCR** **(Ct)**	**IHC Score**	**rRT-PCR** **(Ct)**	**IHC Score**	**Predominant IHC Positive Cell Type/Tissue**
**Lung**	neg	−	neg	n.d.	neg	n.d.	25.03	−/+	39.43	−	Pneumocytes
**Trachea**	37.68	−	neg	n.d.	neg	n.d.	23.83	+	38.46	−	Epithelial cells, macrophages
**Spleen**	neg	n.d.	neg	n.d.	neg	n.d.	23.86	−/+	neg	n.d.	Macrophages
**Duodenum**	neg	n.d.	neg	n.d.	neg	n.d.	25.03	−/+	neg	−	Epithelium
**Pancreas**	neg	n.d.	neg	n.d.	neg	n.d.	24.03	−/+	38.31	−	Acinar necrotic epithelium
**Cecal tonsils**	neg	n.d.	neg	n.d.	neg	n.a.	24.05	+	neg	n.d.	Debris in the crypts, epithelium, necrotic area, endothelium
***Bursa of*** ***Fabricius***	neg	n.d.	neg	n.d.	neg	n.a.	22.25	−	neg	n.a.	n.a.
**Brain**	neg	n.d.	neg	n.d.	neg	n.d.	19.88	+	neg	n.d.	Neurons, glial cells, ependymal

BRL, broiler; rRT-PCR, Real-Time RT-PCR; Ct, cycle threshold; IHC score, − (negative), −/+ (barely/occasional presence of immunolabelled cells), ++ (small number of cells), +++ (moderate number of positive cells), ++++ (numerous positive cells), +++++ (widespread immunolabelling); neg, negative; n.d., not done; n.a., not applicable.

## Data Availability

Not applicable.
